# Validation of a Thai semiquantitative food frequency questionnaire (semi-FFQ) for people at risk of metabolic syndrome

**DOI:** 10.1186/s41043-023-00353-x

**Published:** 2023-02-23

**Authors:** Nattvara Nirdnoy, Kitti Sranacharoenpong, Apinya Surawit, Bonggochpass Pinsawas, Pichanun Mongkolsucharitkul, Tanyaporn Pongkunakorn, Thamonwan Manosan, Suphawan Ophakas, Sophida Suta, Sureeporn Pumeiam, Korapat Mayurasakorn, Siriraj Health Study Group, Winai Ratanasuwan, Mayuree Homsanit, Keerati Charoencholvanich, Bhoom Suktitipat, Chalermchai Mitrpant, Manop Pithukpakorn, Prapat Suriyaphol, Rungroj Krittayaphong, Prasert Auewarakul, Boonrat Tassaneetritap, Naravat Poungvarin

**Affiliations:** 1 Institute of Nutrition, Mahidol University, 999 Phuttamonthon 4 Road, Salaya, Phuttamonthon, Nakhon Pathom, 73130 Thailand; 2 Population Health and Nutrition Research Group, Faculty of Medicine Siriraj Hospital, Mahidol University, 2 Wanglang Road, Siriraj, Bangkoknoi, Bangkok, 10700 Thailand; 3 Faculty of Medicine Siriraj Hospital, Mahidol University, 2 Wanglang Road, Siriraj, Bangkoknoi, Bangkok, 10700 Thailand

**Keywords:** Dietary tool, Semiquantitative food frequency questionnaire, Metabolic syndrome, Validity

## Abstract

**Background:**

Food frequency questionnaires (FFQ) are a useful dietary assessment tool to determine relationships between diet and non-communicable diseases (NCDs). Our purpose was to validate a semiquantitative FFQ (semi-FFQ) for Thais at risk of metabolic syndrome (MS).

**Methods:**

The researchers identified 345 men and women aged 30–65 years who were eligible for the study. Ninety-four participants were finally enrolled (54 in a “urine-collection not-required” group and 40 in a “urine collection” group). They were asked to maintain a 4-day food record for 4 weeks and partook in a semi-FFQ interview during week 4. Urine samples and biochemical results related to MS were collected. Validation results were associated with three primary nutrients for MS (sugar, fat, and sodium) and biochemical results (blood glucose, lipid profiles, blood pressure, and 24-h urine sodium).

**Results:**

The biomarker level of each key MS nutrient significantly increased commensurate with rises in semi-FFQ estimated intakes. Correlation coefficients (*r*) were as follows: fasting blood glucose, *r* = 0.221 (fruits) and *r* = 0.229 (desserts); triglycerides, *r* = 0.112 (a la carte-dishes); low-density lipoprotein cholesterol, *r* = 0.205 (rice-with-topping dishes); systolic blood pressure, *r* = 0.272 (snacks) and *r* = 0.190 (a la carte dishes). Fasting blood glucose was a significant biomarker associated with the development of metabolic syndrome (OR 1.42, 95% CI 1.12–1.81). We also found that fat (OR 1.28, 95% CI 1.09–1.89), sodium (OR 1.98, 95% CI 1.05–1.95) and energy (OR 1.09, 95% CI 1.01–1.17) from an a la carte meal were significantly associated with the development of metabolic syndrome.

**Conclusions:**

Thai food has a unique characteristic since it often pairs various ingredients and seasoning in one menu. This semi-FFQ is a tool that offers relatively valid ranking for intake of energy, nutrients, single foods, and mixed dishes based on Thai menus associated with a risk for developing metabolic syndrome and NCDs. Using this tool could help identify unhealthy dietary patterns and help develop recommendations for people at risk with the goal of preventing NCDs.

## Background

Metabolic syndrome is associated with an increased risk in the development of non-communicable diseases such as diabetes mellitus, hypertension, cancer, and cardiovascular diseases [1–3]. In Thailand, non-communicable diseases account for an estimated 74% of deaths [4]. Diet is a main lifestyle-related risk factor of these metabolic diseases. Nevertheless, metabolic syndrome can be prevented by changing eating behavior and lifestyle habits. And dietary assessment has been useful in NCD risk prediction while consuming a healthy nutrient-dense was correlated with a lower risk of death [5]. Due to the uniqueness of Thai food, it is challenging to estimate the usual intake of a single food type or the ingredients of mixed dishes. The amount and the types of food and ingredients consumed is also various among subjects [6]. To improve our understanding of the eating behavior of Thais, we previously developed a 91-item Thai semiquantitative food frequency questionnaire (semi-FFQ) that encompassed single food items and mixed dishes [7]. However, inaccurate dietary assessment may be a serious barrier of understanding the impact of dietary factors on NCDs. Therefore, the validity of the questionnaire needed to be established.

Epidemiological studies have used various reference methods to validate semi-FFQs, such as dietary records or 24-h recall. For example, Kobayashi et al. developed a semi-FFQ for Japanese children in 2011. Four weighted dietary records were conducted once a week, and the research focused on the correlation between the records and the semi-FFQ [8]. Another cohort study carried out in 2009 validated a semi-FFQ using 24-h dietary recalls. The investigators found correlation coefficients between 2 of 3 sets of 24-h dietary recalls and a 204-item semi-FFQ [9]. Rachmah et al. developed a semi-FFQ for sugar intake for Indonesian children. They used food records for six nonconsecutive days as their intake references [10]. In 2020, Mumu et al. in Bangladesh validated a semi-FFQ using a 24-h dietary recall method and the corresponding biological nutritional markers. Their study was the first to validate a semi-FFQ in Bangladesh using multiple measures, and there was acceptable agreement on ranking the dietary intake of the semi-FFQ with some biomarkers [11].

Nevertheless, each of the above methods has limitations. Dietary records and 24-h recall face the problems of respondent burden, self-report bias, and incorrect recall of information from memory [12, 13]. On the other hand, investigators using biomarkers as an alternative need to be aware of the problems presented by improper collection, transportation methods, and confounders [14]. Therefore, researchers should consider the limitations of each method before deciding on one for their studies. The present investigation aimed to validate the semi-FFQ that had been previously developed for Thais at risk of metabolic syndrome by analyzing the relationship between nutrients-derived from the semi-FFQ and biochemical markers.

## Methods

### Study setting and sample selection

**Fig. 1:**
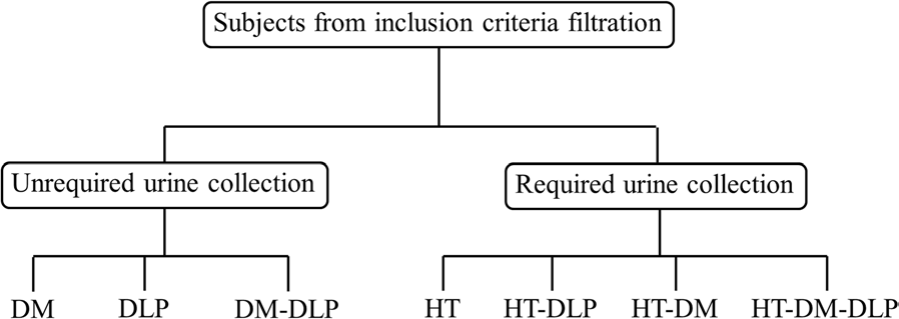
Subject categorization

The validation study was conducted at the Faculty of Medicine Siriraj Hospital, Bangkok, Thailand. The sample population was Siriraj personnel aged 30–65 years who were at risk of metabolic syndrome and had participated in the SIRIRAJ-Health (SI-Health) study. The SI-Health study is a prospective cohort study on non-communicable diseases that began in mid-2017. As of February 2019, its cohort had 4496 Siriraj staff members. The sample selection drew upon data previously obtained for SI-Health. Participants were divided into two main groups according to urine collection requirements: “urine-collection not-required” (a sugar and fat study group) and “urine collection” (a sodium study group). They were then divided into seven minor groups according to the most recent metabolic syndrome criteria of the International Diabetes Federation [3]. They were as follows: (1) dyslipidemia group qualified (DLP); (2) diabetes mellitus group qualified (DM); (3) hypertension group qualified (HT); (4) dyslipidemia and diabetes mellitus group qualified (DM-DLP); (5) hypertension and diabetes mellitus group qualified (HT-DLP); (6) hypertension and diabetes mellitus group qualified (HT-DM); and (7) hypertension, diabetes mellitus, dyslipidemia group qualified (HT-DM-DLP). Figure 1 illustrates the categorization of the subjects.

### Study design and data collection

The validity of the Thai semi-FFQ was assessed by analyzing the relationship between the nutrients derived from the Thai semi-FFQ and the biochemical results. The biochemical results of each participant were sourced from SI-Health, which records data on annual health checks.

The duration of data collection was 4 weeks. Participants were required to attend two interviews conducted in weeks 1 and 4 at Siriraj Medical Research Center, Siriraj Hospital, Thailand. Between these interview sessions, participants could use the social networking application LINE (Line Corporation, Tokyo, Japan) to ask for more information or make an appointment for a face-to-face consultation.

In the first interview session, prospective subjects were provided with documents that described the objectives and procedures of the study. The subjects were asked to sign a consent form if they agreed to participate, and they were informed that they could withdraw at any time during the study. The investigator then explained the data collection process. Subjects in the HT, HT-DLP, HT-DM, and HT-DM-DLP subgroups were asked to undergo a 24-h urine collection. In the second interview session, the participants partook in the Thai semi-FFQ interview carried out by a dietitian.

### 24-h urine collection

**Fig. 2:**
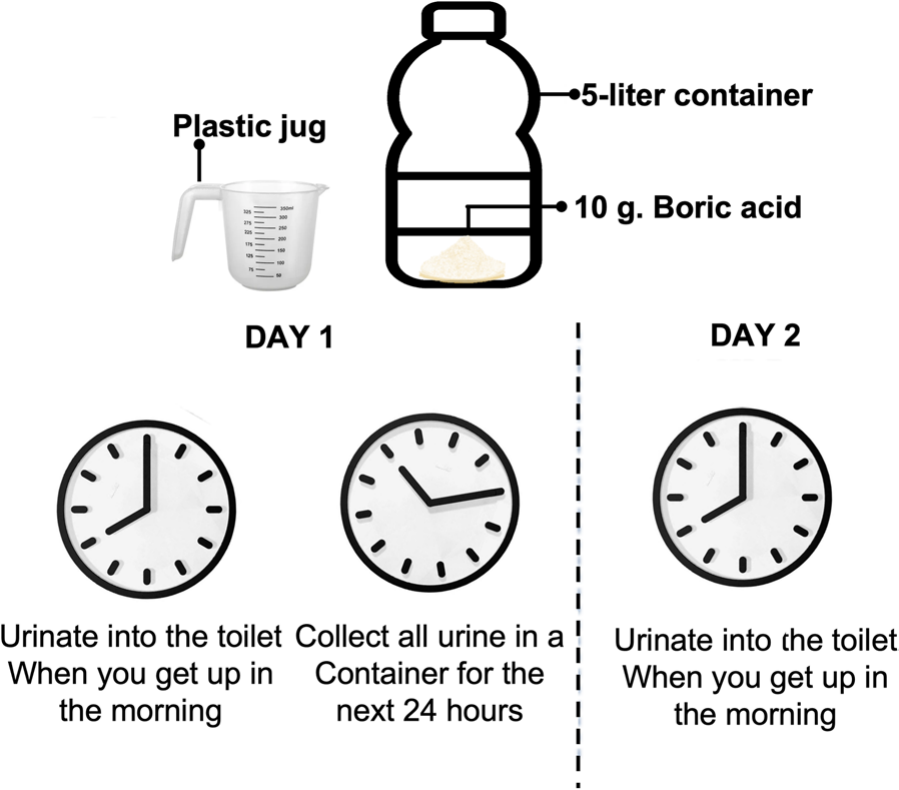
24 h urine collection procedure

Participants in the urine collection group were asked to record the date and time of their first urination in the morning but were instructed not to collect a sample. However, during the following 24 h, all urine was collected. To this end, an investigator provided participants with a plastic jug and a 5-L container, each using 10 g of boric acid as a preservative. The sodium in the urine was later analyzed using an indirect ion-selective electrode (Fig. 2).

### Thai semi-FFQ

**Fig. 3:**
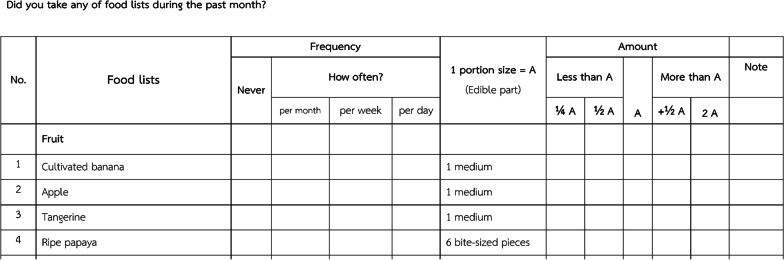
Example of Thai semi-FFQ (English version)

A semi-FFQ was administered in week 4. The Thai semi-FFQ was initially designed for use in the SI-Health study to obtain data on the frequency of food consumption and the amount consumed in 1 month. Methods for development of semi-FFQ were previously described [7]. Briefly, food types and amounts consumed from dietary recall and our observation in a pilot study was ranked by registered nutritionists in gram/unit intake. For food groups, itemized single foods, mixed dishes, sweets, and desserts were classified. We focused on a la carte, noodles, or rice with topping-dish. Each menu was three randomly selected sampling to measure each ingredient. The major ingredients of rice or noodle dishes, as well as meat and vegetable groups of each Thai mixed dish were separated and weighted by a digital kitchen scale (TANITA digital kitchen scale; KD-192, Japan). Nutritive values of all the foods along with their condiments were calculated by Thai food composition software (INMUCAL-v3.2) of the Institute of Nutrition, Mahidol University, Thailand. The nutritive values of food items consisted of energy, total fat, protein, sodium, sugar, dietary fiber, cholesterol, and saturated fat. Nutrient profiling (NP) was used to classify foods and snacks based on their nutritional composition. There were three levels of grading: ‘grade A’ (a suitable nutrient profile with no or low risk for NCDs), ‘grade B’ (unsuitable nutrient profile with medium risk for NCDs), and ‘grade C’ (unsuitable nutrient profile with high risk for NCDs). Mean values of each nutrient in every food items were calculated before using the NP criteria. The eating behavior questionnaires were also included for supporting dietary patterns. Finally, three patterns of FFQ that emphasized on the amounts of sugar, sodium and fat was identified since these nutrients were strongly related to MS. The food menus and serving sizes were added in the FFQ. The content validity index (CVI) was evaluated by qualified experts and nutritionists. The CVI consists of a 4-point rating scale (1 = not relevant, 4 = very relevant). An acceptable level of content validity should be greater than 0.8. After the FFQ development, three experts from Institute of Nutrition, Mahidol University, were asked for reviewing the FFQ. The Thai semi-FFQ has 97 food items: 18 tropical fruits, ten beverages, 29 snacks and desserts, 23 mixed dishes (a la carte and noodles), and 17 rice-with-topping dishes. A 5-level scale is used for serving size; the serving size unit is the “household unit” of the Thai food-based dietary guideline [15]. The frequency response options are open-ended, and the choices are “never,” “x times per month,” “x times per week,” and “x times per day” (Fig. 3). The questionnaire was completed during the interview conducted by a dietitian.

### Data preparation

The Thai food composition software INMUCAL-Nutrients V.4.0, Database NB.4 (Institute of Nutrition, Mahidol University, Nakhon Pathom, Thailand), was used to calculate the nutrient intake from the Thai semi-FFQ. Nine nutrients were analyzed: energy (kcal), carbohydrate (g), total fat (g), protein (g), sodium (mg), sugar (g), dietary fiber (g), cholesterol (mg), and saturated fat (g).

Biochemical results related to metabolic syndrome criteria were obtained from the SI-Health cohort study. They were fasting blood glucose (FBG), glycated hemoglobin (HbA1c), cholesterol level, triglyceride level (TG), high-density lipoprotein cholesterol (HDL-C), low-density lipoprotein cholesterol (LDL-C), systolic blood pressure (SBP), and diastolic blood pressure (DBP). These results were collected and analyzed in the same day as the subjects participated in annual health checkup between 2019 and 2020, which was the same time as we conducted the semi-FFQ.

To overcome the limitation of correlation analysis, reducing the variation between data points was considered. Consequently, “popular foods” were selected from the items in the Thai semi-FFQ; the resulting list consisted of foods consumed by at least one-third of the participants.

### Statistical analysis

Statistical analyses were performed using IBM SPSS Statistics for Windows, version 22.0 (IBM Corp., Armonk, NY, USA). General data and health information are presented as mean, median, standard deviation, frequency, and percentage. The correlations between the focus nutrients of the Thai semi-FFQ and biochemical results (both blood and urine) were measured using Spearman’s rank correlation or Pearson’s correlation for the normally distributed data (Table 1). Two-tailed statistical analyses were conducted at a probability (*P*) value of 0.05 to determine statistical significance. To decrease the variations between data, bootstrap resampling was used. Risk factors for the development of metabolic syndrome were analyzed using a multivariable logistic regression.

**Table 1:** Data analysis for relationship between nutrients and biochemistry results

Nutrients from semi-FFQ	Biochemistry results	Data analysis
Sodium	24-h urine sodium	Normal distribution: Pearson’s correlation
	Systolic blood pressure; SBP	
	Diastolic blood pressure; DBP	
Sugar	Fasting blood glucose; FBG	Non-normal distribution: Spearman’s rank correlation
	Glycated hemoglobin; HbA1c	
Fat	Triglyceride level; TG	
	High-density lipoproteins; HDL-C	

## Results

### Participants in this study

**Fig. 4:**
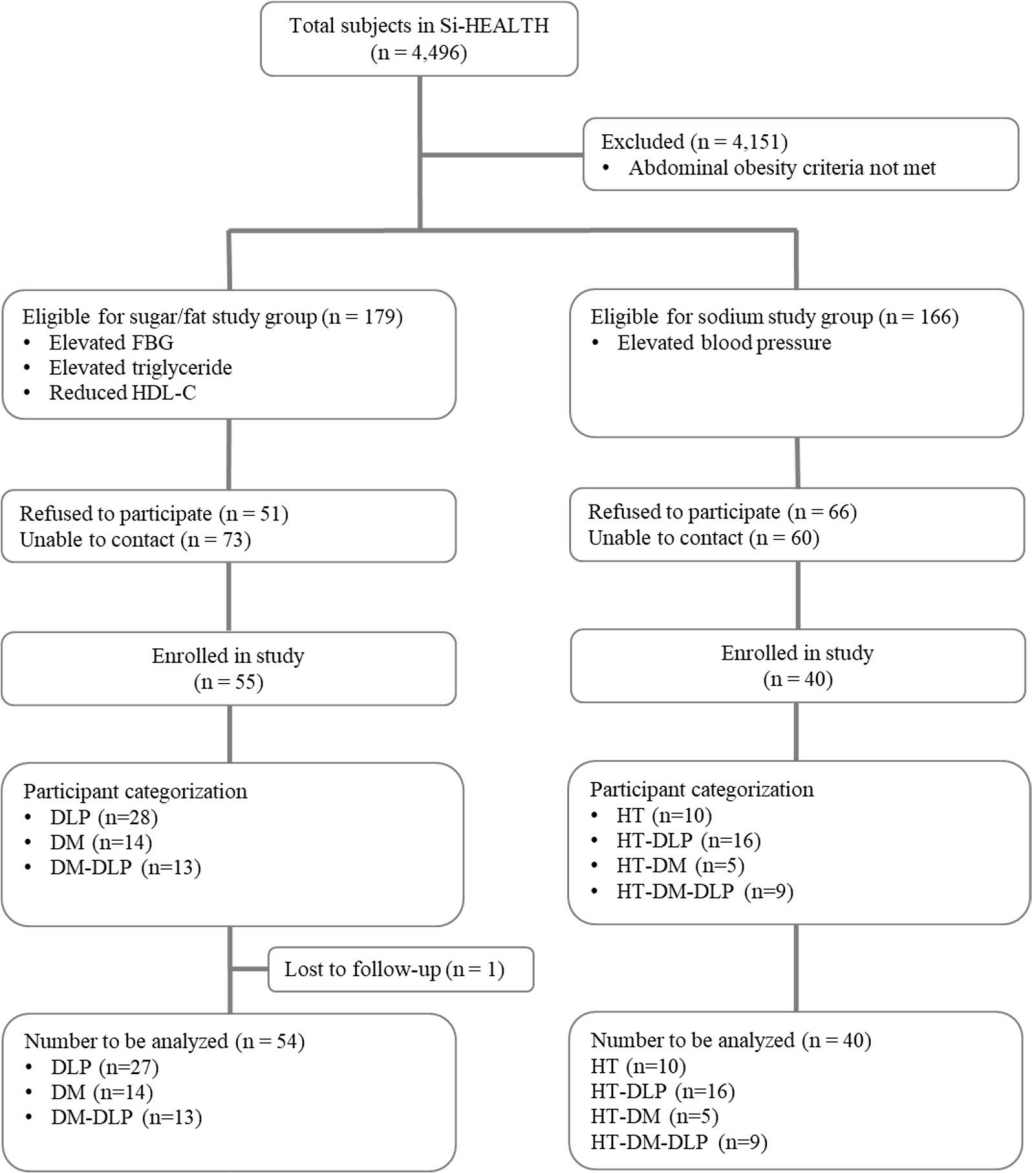
Subject recruitment and allocation in this study

Of the 4496 people in the SI-Health study cohort, 345 were eligible for enrollment in the current investigation. The eligible participants were divided into two main groups based on their SI-Health results: a sugar and fat study group (the urine-collection not-required group) and a sodium study group (the urine collection group). Phone recruitment was conducted; 117 people declined to participate, and 133 could not be contacted. Eventually, 95 participants were enrolled. During the study, one person in the DLP group was lost to follow-up. Therefore, the number of participants analyzed in this study was 94 (Fig. 4). Ninety-four participants were classified into seven subgroups: DLP (*n* = 27), DM (*n* = 14), HT (*n* = 10), DM-DLP (*n* = 13), HT-DLP (*n* = 16), HT-DM (*n* = 5), and HT-DM-DLP (*n* = 9). The mean age of the validity study participants was 40.13 ± 7.13 years. Women predominated in all subgroups except the HT-DM-DLP group, which had an equal number of men and women. Almost 80% of the participants had a BMI greater than 25 kg/m^2^ (Table 2).

**Table 2:** Characteristics of participants

% or Mean ± SD	DLP (*n* = 27)	DM (*n* = 14)	HT (*n* = 10)	DM-DLP (*n* = 13)	HT-DLP (*n* = 16)	HT-DM (*n* = 5)	HT-DM-DLP (*n* = 9)	Total (*N* = 94)
Gender (%)								
Male	18.52	0.00	20.00	30.77	43.75	20.00	66.67	26.32
Female	81.48	100.00	80.00	69.23	56.25	80.00	33.33	72.63
Workout (%)								
Rarely	66.67	71.43	60.00	76.92	68.75	60.00	33.33	64.21
Sometimes (1/week)	29.63	28.57	30.00	23.08	31.25	20.00	55.56	30.53
Often (2–3/week)	3.70	0.00	10.00	0.00	0.00	20.00	11.11	4.21
Usually (> 3/week)	0.00	0.00	0.00	0.00	0.00	0.00	0.00	0.00
Smoking (%)								
No	92.59	100.00	80.00	92.31	87.50	100.00	55.56	87.37
Stop	7.41	0.00	20.00	7.69	12.50	0.00	44.44	11.58
Yes	0.00	0.00	0.00	0.00	0.00	0.00	0.00	0.00
Alcohol consumption (%)								
Never	48.15	57.14	50.00	46.15	56.25	20.00	33.33	47.37
Stop	11.11	0.00	0.00	0.00	0.00	20.00	0.00	4.21
Occasionally (< 1/month)	25.93	28.57	30.00	53.85	31.25	40.00	22.22	31.58
Sometimes (1/month)	14.81	14.29	10.00	0.00	12.50	20.00	44.44	14.74
Usually (> 1/week)	0.00	0.00	10.00	0.00	0.00	0.00	0.00	1.05
Age (years)								
< 35	33.33	0.00	30.00	15.38	37.50	20.00	44.44	26.32
35–45	44.44	64.29	20.00	69.23	43.75	60.00	44.44	48.42
45–55	18.52	35.71	50.00	15.38	18.75	20.00	11.11	23.16
55–65	3.70	0.00	0.00	0.00	0.00	0.00	0.00	1.05
Mean age (years)	39.81 ± 7.04	43.36 ± 5.31	40.10 ± 10.75	40.77 ± 7.08	38.44 ± 6.88	41.00 ± 6.12	37.67 ± 6.26	40.13 ± 7.13
BMI (kg/m^2^)								
18.5–23	14.81	7.14	10.00	0.00	0.00	20.00	0.00	7.37
23–25	0.00	14.29	30.00	7.69	18.75	20.00	11.11	11.58
25–30	59.26	57.14	40.00	76.92	56.25	40.00	44.44	55.79
> 30	25.93	21.43	20.00	15.38	25.00	20.00	44.44	24.21
Mean BMI (kg/m^2^)	28.06 ± 3.84	27.15 ± 3.00	26.80 ± 3.59	28.12 ± 3.55	28.35 ± 2.98	28.30 ± 6.98	31.40 ± 5.22	28.17 ± 3.92
WC-Ht/2 ratio								
< 1.25times	77.78	35.71	100.00	76.92	75.00	80.00	55.56	70.53
1.25–1.5times	22.22	64.29	0.00	23.08	25.00	20.00	33.33	26.32
> 1.5times	0.00	0.00	0.00	0.00	0.00	0.00	11.11	2.11
SBP (mmHg)								
< 120	62.96	64.29	0.00	53.85	0.00	0.00	0.00	34.74
120–130	37.04	35.71	10.00	46.15	0.00	20.00	0.00	24.21
130–140	0.00	0.00	80.00	0.00	68.75	40.00	44.44	26.32
140–150	0.00	0.00	10.00	0.00	25.00	40.00	11.11	8.42
> 150	0.00	0.00	0.00	0.00	6.25	0.00	44.44	5.26
Mean SBP (mmHg)	115.44 ± 8.94	115.64 ± 9.64	133.90 ± 4.41	117.85 ± 7.26	136.13 ± 5.25	133.80 ± 8.17	146.11 ± 11.40	125.22 ± 13.58
DBP (mmHg)								
< 80	81.48	85.71	30.00	76.92	31.25	40.00	11.11	57.89
80–85	18.52	14.29	30.00	15.38	37.50	40.00	22.22	21.05
85–90	0.00	0.00	10.00	7.69	25.00	20.00	11.11	9.47
90–95	0.00	0.00	30.00	0.00	6.25	0.00	22.22	6.32
> 95	0.00	0.00	0.00	0.00	0.00	0.00	33.33	4.21
Mean DBP (mmHg)	70.78 ± 7.60	72.29 ± 8.53	83.10 ± 7.32	72.38 ± 9.26	81.75 ± 7.02	82.20 ± 7.05	92.00 ± 19.91	77.05 ± 11.58
FBG (mmol/L)								
< 5.56	100.00	0.00	100.00	0.00	100.00	0.00	0.00	55.79
5.56–6.94	0.00	100.00	0.00	84.62	0.00	100.00	66.67	37.89
6.94–10.00	0.00	0.00	0.00	15.38	0.00	0.00	33.33	5.26
Mean FBG (mmol/L)	4.98 ± 0.27	5.81 ± 0.31	4.92 ± 0.23	6.18 ± 0.51	5.09 ± 0.39	5.79 ± 0.12	6.51 ± 1.31	5.42 ± (0.50)
Cholesterol (mmol/L)								
< 5.17	48.15	50.00	70.00	30.77	37.50	40.00	55.56	46.32
5.17–6.21	33.33	42.86	20.00	46.15	37.50	20.00	22.22	33.68
> 6.21	18.52	7.14	10.00	23.08	25.00	40.00	22.22	18.95
Mean cholesterol (mmol/L)	5.21 ± 0.91	5.15 ± 0.89	5.10 ± 0.79	5.63 ± 1.04	5.31 ± 0.92	5.64 ± 0.82	5.08 ± 1.30	5.18 ± (0.67)
Triglyceride (mmol/L)								
< 1.69	14.81	100.00	100.00	23.08	0.00	100.00	22.22	40.00
1.69–2.26	62.96	0.00	0.00	61.54	62.50	0.00	33.33	40.00
2.26–5.65	18.52	0.00	0.00	15.38	37.50	0.00	44.44	17.89
> 5.65	3.70	0.00	0.00	0.00	0.00	0.00	0.00	1.05
Mean triglyceride (mmol/L)	2.36 ± 1.41	1.09 ± 0.39	1.01 ± 0.25	2.10 ± 0.75	2.41 ± 0.74	1.15 ± 0.29	2.19 ± 0.90	1.80 ± (1.05)
HDL-C (mmol/L)*								
Optimal	18.52	21.43	20.00	30.77	6.25	0.00	0.00	15.79
Borderline	29.63	71.43	60.00	30.77	43.75	100.00	33.33	45.26
Low	51.85	7.14	20.00	38.46	50.00	0.00	66.67	37.89
Mean HDL-C (mmol/L)	1.42 ± 0.65	1.81 ± 0.60	1.65 ± 0.31	1.65 ± 0.93	1.20 ± 0.32	1.64 ± 0.16	1.12 ± 0.14	1.40 ± (0.21)
LDL-C (mmol/L)								
< 3.36	74.07	64.29	80.00	46.15	56.25	60.00	77.78	65.26
3.36–4.14	11.11	28.57	10.00	23.08	25.00	20.00	11.11	15.79
4.14–4.91	14.81	7.14	10.00	23.08	18.75	20.00	11.11	13.68
> 4.91	0.00	0.00	0.00	7.69	0.00	0.00	0.00	4.21
Mean LDL-C (mmol/L)	2.69 ± 1.01	2.87 ± 0.88	3.09 ± 0.52	2.99 ± 1.37	3.08 ± 1.01	3.56 ± 0.92	3.12 ± 1.08	3.10 ± (0.95)
HbA1c (mmol/mol)								
< 38.80	66.67	21.43	40.00	7.69	56.25	0.00	33.33	40.00
38.80–47.54	33.33	64.29	60.00	53.85	43.75	100.00	33.33	48.42
> 47.54	0.00	14.29	0.00	38.46	0.00	0.00	33.33	10.53
Mean HbA1c (mmol/mol)	36.07 ± 19.02	41.42 ± 18.14	39.34 ± 20.87	44.92 ± 18.36	36.94 ± 19.45	40.77 ± 22.08	46.78 ± 8.09	39.89 ± 16.39

*HDL-C: Optimal (> 1.55 mmol/L for male and > 1.81 mmol/L for female), Borderline (1.03–1.55 mmol/L for male and 1.29–1.81 mmol/L for female), Low (< 1.03 mmol/L for male and < 1.29 mmol/L for female)

### Data preparation

**Fig. 5:**
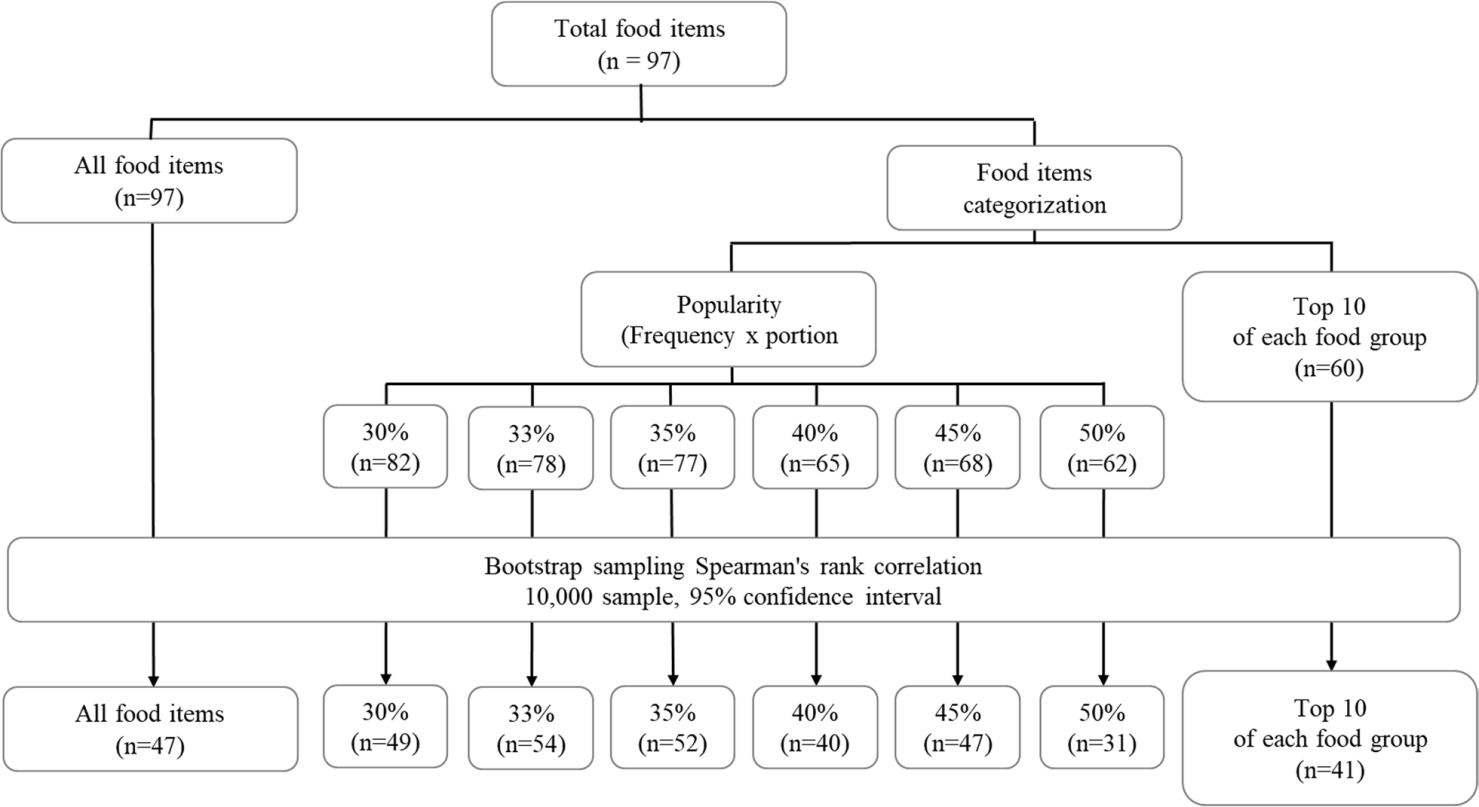
Data preparation flowchart

Before analysis, we reviewed the data to establish the best validation model that would provide appropriate correlation coefficient values between food items and reference indicators. We focused on the frequency of consumption of each food item and determined its popularity by multiplying that frequency by the portion size. We set distinct levels of popularity, signified by 30%, 33%, 35%, 40%, 45%, and 50% of participants consuming a food item. For example, 57% of DM subjects consumed pasteurized milk, which meant that pasteurized milk was selected for the 50% popularity level but not the 45% popularity level (Fig. 5). After performing statistical analyses, the value of 33% gave the highest number of “popular” food items compared with other procedures. Consequently, we used the 33% cutoff for popularity to analyze the correlation coefficient values.

### Statistical analysis

The bootstrap method was conducted to find a correlation between crucial nutrients for metabolic syndrome (sugar, fat, and sodium) of each food group and biochemical results (FBG, HbA1c, TG, cholesterol, HDL-C, LDL-C, SBP, DBP, and urine sodium). Spearman’s correlation was selected. The popular food items of each food group were calculated by frequency multiplied by portion size. The popular items in each food group are listed in Table 3.

**Table 3:** Popular food items from Thai semi-FFQ

No. FFQ	Sugar and fat group	No. FFQ	Sodium group
1	Cultivated banana	1	Cultivated banana
2	Apple	2	Apple
3	Tangerine	3	Tangerine
4	Ripe papaya	4	Ripe papaya
5	Pineapple	5	Pineapple
6	Young coconut juice	6	Young coconut juice
7	Watermelon	7	Watermelon
9	Unripe mango	9	Unripe mango
15	Grape	15	Grape
16	Langsat	17	Strawberry
18	Guava	18	Guava
19	Iced coffee	19	Iced coffee
20	Concentrated flavored syrup soda	20	Concentrated flavored syrup soda
21	Fruit smoothie with syrup	21	Fruit smoothie with syrup
22	Pasteurized milk	22	Pasteurized milk
24	Soy milk	24	Soy milk
25	Yogurt	25	Yogurt
26	Fermented milk (ex. Yakult)	26	Fermented milk (ex. Yakult)
27	Carbonated soft drink	27	Carbonated soft drink
28	Energy drink/mineral drink	29	Deep fried pork balls/sausages/nuggets
29	Deep fried pork balls/sausages/nuggets	30	Steamed pork balls/sausages
30	Steamed pork balls/sausages	31	Deep fried wonton/Chinese donut
31	Deep fried wonton/Chinese donut	32	Pork satay/grilled pork
32	Pork satay/grilled pork	33	Fresh vegetable rice wraps
34	Steamed bun (Sa-la-pao)	34	Steamed bun (Sa-la-pao)
35	Chinese dumpling (Ka-nom-jeeb)	35	Chinese dumpling (Ka-nom-jeeb)
37	Coated peanuts	36	Tapioca pork/Thai steamed rice dumpling (Kow Griep Pak Mor)
38	Flavored snacks (Potatoes chips)	37	Coated peanuts
39	Sweet flavored snacks (caramel snacks)	38	Flavored snacks (Potatoes chips)
49	Thai deep-fried sweet potato ball	39	Sweet flavored snacks (caramel snacks)
56	Sweet puff pastry	49	Thai deep-fried sweet potato ball
43	Steamed Thai dessert (ex. Ka-nom-chan)	51	Pancake roll with minced pork and egg
44	Coconut rice pancake (Ka-nom-Krok)	57	Puff pastry, bakery products
52	Flavored ice-cream (ex. Vanilla)	43	Steamed Thai dessert (ex. Ka-nom-chan)
53	Coconut milk ice-cream	44	Coconut rice pancake (Ka-nom-Krok)
55	Butter cake, Cake	47	Lod Chong (flour in coconut milk)
58	Thai rice topped with stir-fried pork and hot basil	48	Grass jelly in milk (Chao-Guay)
60	Pork blood cube soup with Thai rice	52	Flavored ice-cream (ex. Vanilla)
61	Pork congee	55	Butter cake, Cake
62	Fried rice with meat	58	Thai rice topped with stir-fried pork and hot basil
63	Steamed chicken and rice	60	Pork blood cube soup with Thai rice
66	Thai rice topped with stir-fried crispy pork and kale	61	Pork congee
67	Thai rice topped with stir-fried mixed vegetable in oyster sauce	62	Fried rice with meat
68	Thai spicy shrimp-paste fried rice	63	Steamed chicken and rice
69	Vietnamese rice noodles soup	64	Fried chicken and rice
70	Egg noodles soup with roasted pork	65	Thai rice topped with stir-fried pork, garlic and pepper
71	Egg noodles with roasted pork	67	Thai rice topped with stir-fried mixed vegetable in oyster sauce
72	Noodles with fish ball and red sauce (Yen-Ta-Four) (without soup/soup)	68	Thai spicy shrimp-paste fried rice
73	Noodles with pork blood and herb (without soup/soup)	69	Vietnamese rice noodles soup
74	Fried noodles in gravy with meat	70	Egg noodles soup with roasted pork
75	Stir-fried noodles in soy sauce with meat (Pad-See-Ew)	71	Egg noodles with roasted pork
76	Thai fried noodles (Pad-Thai)	72	Noodles with fish ball and red sauce (Yen-Ta-Four) (without soup/soup)
77	Thai-style suki with mung bean noodles	73	Noodles with pork blood and herb (without soup/soup)
78	Spicy noodles salad (Yum mama)	74	Fried noodles in gravy with meat
79	Rice noodles in spicy fish paste curry (Kanom-Jeen)	75	Stir-fried noodles in soy sauce with meat (Pad-See-Ew)
81	Tofu, vegetable and minced pork in clear soup	76	Thai fried noodles (Pad-Thai)
82	Coconut milk curry (ex. chicken green curry)	77	Thai-style suki with mung bean noodles
83	Thai soup (ex. Kaeng Liang, Kaeng Pa)	78	Spicy noodles salad (Yum mama)
84	Egg and pork with sweet brown soup (Kai-Pa-Lo)	79	Rice noodles in spicy fish paste curry (Kanom-Jeen)
85	Stir-fried minced pork and basil leaves (Ka-Prow-Moo)	81	Tofu, vegetable and minced pork in clear soup
86	Stir-fried crispy catfish with chili paste	82	Coconut milk curry (ex. chicken green curry)
87	Stir-fried mixed vegetable with oyster sauce	83	Thai soup (ex. Kaeng Liang, Kaeng Pa)
88	Stir-fried pumpkin with egg	84	Egg and pork with sweet brown soup (Kai-Pa-Lo)
89	Fried pork patty	85	Stir-fried minced pork and basil leaves (Ka-Prow-Moo)
90	Thai-style omelet	86	Stir-fried crispy catfish with chili paste
91	Fried egg	87	Stir-fried mixed vegetable with oyster sauce
92	Boiled egg	88	Stir-fried pumpkin with egg
93	Salad	89	Fried pork patty
94	Salad cream	90	Thai-style omelet
		91	Fried egg
		92	Boiled egg
		93	Salad
		94	Salad cream

The validity results for sugar, fat, and sodium among the food groups and the biochemical results are presented in Table 4. Spearman’s rank correlation coefficient found the following ranges: sugar, 0.04 to 0.265; fat, 0.03 to 0.260; and sodium, 0.01 to 0.272. The correlation coefficient with/without bootstrap resampling was not different. The highest correlations were obtained for the sugars in tropical fruits and FBG, with 0.265. The lowest correlations were found for the sodium in the dessert and DBP, with a value of 0.01.

**Table 4:** Compare between correlation and bootstrapping a correlation of nutrient intake from semi-FFQ and biochemical results

Food group	Variables		Correlation		Bootstrapping a correlation			
	Nutrient	Biochemical results	Correlation coefficient	*P* value	Correlation coefficient	95% CI	Bias factor	*P* value
Fruit	Sugar (g)	FBG	0.265**	0.01	0.221**	0.15, 0.54	0	0.00
		HbA1c	0.18	0.09	0.18	− 0.02, 0.36	0	0.09
Beverage	Sugar (g)	FBG	− 0.04	0.74	− 0.14	− 0.42, 0.15	0	0.52
		HbA1c	− 0.15	0.14	− 0.15	− 0.36, 0.05	0	0.14
Snack	Sugar (g)	FBG	− 0.08	0.46	− 0.11	− 0.29, 0.19	0	0.46
		HbA1c	− 0.05	0.63	− 0.05	− 0.26, 0.18	0	0.63
Snack	Fat (g)	TG	0.18	0.09	0.13	− 0.23, 0.45	0	0.21
		CHOL	− 0.08	0.45	− 0.09	− 0.22, 0.31	0	0.37
		HDL-C	− 0.15	0.17	− 0.14	− 0.17, 0.10	0	0.19
		LDL-C	0.09	0.31	0.15	− 0.23, 0.34	0	0.18
Snack	Sodium (mg)	SBP	0.272**	0.01	0.272**	0.08, 0.44	0	0.01
		DBP	0.19	0.07	0.19	− 0.01, 0.37	0	0.07
		Urine sodium	0.14	0.18	0.12	− 0.07, 0.31	0	0.23
Dessert	Sugar (g)	FBG	0.235*	0.04	0.229**	0.15, 0.48	0.00^║^	0.01
		HbA1c	0.223*	0.03	0.223*	0.04, 0.39	0	0.03
Dessert	Fat (g)	TG	0.07	0.55	− 0.08	− 0.49, 0.12	0	0.45
		CHOL	− 0.15	0.16	− 0.10	− 0.27, 0.19	0	0.81
		HDL-C	0.14	0.19	0.18	− 0.11, 0.35	0	0.19
		LDL-C	− 0.11	0.32	− 0.15	− 0.24, 0.19	0	0.73
Dessert	Sodium (mg)	SBP	0.03	0.77	0.03	− 0.16, 0.23	0	0.77
		DBP	0.01	0.92	0.01	− 0.19, 0.20	0	0.92
		Urine sodium	− 0.03	0.76	− 0.05	− 0.24, 0.15	0	0.66
A la carte	Fat (g)	TG	− 0.214*	0.05	− 0.112*	0.09, 0.41	0.00^║^	0.02
		CHOL	0.11	0.31	0.07	− 0.12, 0.38	0	0.64
		HDL-C	− 0.12	0.27	− 0.16	− 0.35, 0.14	0	0.13
		LDL-C	0.03	0.81	0.02	− 0.11, 0.29	0	0.88
A la carte	Sodium (mg)	SBP	0.206*	0.05	0.19	0.00, 0.39	0	0.05
		DBP	0.08	0.44	0.08	− 0.13, 0.29	0	0.44
		Urine sodium	0.14	0.18	0.11	− 0.09, 0.33	0	0.22
Noodles	Fat (g)	TG	0.14	0.20	0.21*	− 0.15, 0.49	0	0.05
		CHOL	0.05	0.67	0.02	− 0.20, 0.19	0	0.86
		HDL-C	− 0.17	0.11	− 0.14	− 0.22, 0.16	0	0.19
		LDL-C	0.04	0.70	0.03	− 0.13, 0.22	0	0.79
Noodles	Sodium (mg)	SBP	0.09	0.4	0.09	− 0.12, 0.29	0	0.40
		DBP	0.12	0.25	0.12	− 0.07, 0.30	0	0.25
		Urine sodium	0.10	0.36	0.08	− 0.12, 0.28	0	0.43
Toppings	Fat (g)	TG	0.06	0.56	0.242*	0.09, 0.34	0	0.02
		CHOL	0.03	0.81	0.04	− 0.09, 0.39	0	0.75
		HDL-C	0.03	0.78	0.01	− 0.20, 0.25	0	0.58
		LDL-C	0.260*	0.01	0.205*	0.09, 0.35	0	0.05
Toppings	Sodium (mg)	SBP	0.05	0.66	0.05	− 0.15, 0.24	0	0.66
		DBP	0.08	0.42	0.08	− 0.12, 0.29	0	0.42
		Urine sodium	− 0.04	0.70	− 0.06	− 0.26, 0.16	0	0.60

^*^Correlation is significant at the 0.05 level (2-tailed)

^**^Correlation is significant at the 0.01 level (2-tailed)

^║^Quantities of blood test results and nutrients were log-transformed where the test of normality was significant

Spearman’s correlation coefficient produced significant values for the sugars in tropical fruits, sugar in desserts, FBG, and HbA1c. The sugars in tropical fruits and desserts compared with FBG ranged from 0.235 to 0.265; when compared with HbA1c, they ranged from 0.180 to 0.223. The bootstrapping correlation coefficient values for resampling the data were 0.221 for the sugars in tropical fruits (FBG), 0.229 for sugar in desserts (FBG), 0.180 for the sugars in tropical fruits (HbA1c), and 0.223 for sugar in desserts (HbA1c). There were no significant correlations between the sugar content of beverages or snacks and FBG and HbA1c.

Spearman’s correlation coefficient produced significant values for fat in a la carte dishes, fat in rice-with-topping dishes and TG, cholesterol, HDL-C, and LDL-C. The fat in a la carte dishes and rice-with-topping dishes compared with TG ranged from 0.060 to 0.214; compared with LDL-C, they ranged from 0.030 to 0.260. The bootstrapping correlation coefficient values for resampling the data were 0.112 (TG and fat in a la carte dishes), 0.242 (TG and fat in rice-with-topping dishes), 0.020 (LDL-C and fat in a la carte dishes), and 0.205 (LDL-C and fat in rice-with-topping dishes). There were no significant correlations between the fat content of snacks, desserts, and noodles and TG, cholesterol, HDL-C, or LDL-C.

Spearman’s correlation coefficient produced statistically significant values for the sodium in snacks and a la carte dishes and SBP. The sodium in snacks and a la carte dishes compared with SBP ranged from 0.206 to 0.272. The bootstrapping correlation coefficient values for resampling the data were 0.190 for sodium in snacks (LDL-C) and 0.272 for sodium in a la carte dishes (LDL-C). There were no significant correlations between the sodium content of snacks or a la carte dishes and DBP and urinary sodium. Table 5 shows the correlation between the energy intake (kcal) from semi-FFQ and the biochemical results. The correlation coefficients of energy intake from desserts, TG, and cholesterol were significantly correlated. The bootstrapping correlation values were − 0.239 (desserts and TG) and 0.235 (desserts and cholesterol). The Thai semi-FFQ consists of 54 items with significant biochemical results, as summarized in Table 5.

**Table 5:** Compare between correlation and bootstrapping a correlation of energy intake from semi-FFQ and biochemical results

Food group	Variables		Correlation		Bootstrapping a correlation			
	Nutrient	Biochemical results	Correlation coefficient	*P* value	Correlation coefficient	95% CI	Bias factor	*P* value
Fruit	Energy (kcal)	FBG	− 0.03	0.76	− 0.07	− 0.29, 0.16	0	0.50
		HbA1c	0.03	0.82	0.03	− 0.21, 0.27	0	0.82
Beverage	Energy (kcal)	FBG	− 0.05	0.66	− 0.02	− 0.31, 0.10	0	0.86
		HbA1c	− 0.07	0.55	− 0.07	− 0.32, 0.19	0	0.55
Snack	Energy (kcal)	FBG	− 0.16	0.12	− 0.12	− 0.30, 0.17	0	0.26
		HbA1c	− 0.05	0.68	− 0.05	− 0.23, 0.20	0	− 0.05
Snack	Energy (kcal)	TG	0.21	0.06	0.238*	0.11, 0.35	0	0.01
		CHOL	− 0.08	0.46	− 0.10	− 0.29, 0.16	0	0.36
		HDL-C	− 0.17	0.11	− 0.15	− 0.25, 0.25	0	0.15
		LDL-C	− 0.10	0.33	− 0.14	− 0.31, 0.21	0	0.18
Snack	Energy (kcal)	SBP	− 0.07	0.57	− 0.07	− 0.28, 0.15	0	0.57
		DBP	0.04	0.74	0.04	− 0.18, 0.25	0	0.74
		Urine sodium	0.07	0.55	0.07	− 0.17, 0.30	0	0.54
Dessert	Energy (kcal)	FBG	− 0.03	0.76	− 0.04	− 0.31, 0.15	0	0.69
		HbA1c	− 0.08	0.52	− 0.08	− 0.29, 0.23	0	0.52
Dessert	Energy (kcal)	TG	− 0.223*	0.04	− 0.239*	− 0.44, − 0.09	0	0.02
		CHOL	0.16	0.14	0.235*	0.15, 0.52	0.00^║^	0.03
		HDL-C	0.10	0.37	0.11	− 0.36, 0.47	0	0.28
		LDL-C	− 0.13	0.24	− 0.13	− 0.25, 0.23	0	0.22
Dessert	Energy (kcal)	SBP	− 0.05	0.67	− 0.05	− 0.30, 0.21	0	0.67
		DBP	− 0.02	0.89	− 0.02	− 0.27, 0.23	0	0.89
		Urine sodium	0.01	0.94	0.03	− 0.22, 0.27	0	0.82
A la carte	Energy (kcal)	TG	0.15	0.15	0.11	− 0.17, 0.28	0	0.29
		CHOL	− 0.05	0.61	− 0.03	− 0.24, 0.16	0	0.81
		HDL-C	0.04	0.74	0.04	− 0.11, 0.30	0	0.73
		LDL-C	− 0.12	0.27	− 0.08	− 0.12, 0.09	0	0.44
A la carte	Energy (kcal)	SBP	0.04	0.75	0.04	− 0.20, 0.26	0	0.75
		DBP	0.02	0.84	0.02	− 0.22, 0.26	0	0.84
		Urine sodium	− 0.01	0.96	0.00	− 0.24, 0.24	0	0.97
Noodles	Energy (kcal)	TG	− 0.14	0.17	− 0.20	− 0.33, 0.09	0	0.09
		CHOL	− 0.06	0.59	− 0.01	− 0.22, 0.11	0	0.93
		HDL-C	0.13	0.21	0.16	− 0.29, 0.26	0	0.13
		LDL-C	− 0.06	0.59	− 0.06	− 0.20, 0.28	0	0.58
Noodles	Energy (kcal)	SBP	− 0.01	0.92	− 0.01	− 0.24, 0.21	0	0.92
		DBP	0.02	0.85	0.02	− 0.22, 0.26	0	0.85
		Urine sodium	0.04	0.72	0.05	− 0.18, 0.28	0	0.67
Toppings	Energy (kcal)	TG	0.234*	0.03	0.213*	0.22, 0.37	0	0.04
		CHOL	0.03	0.81	0.03	− 0.24, 0.21	0	0.80
		HDL-C	0.02	0.98	0.03	− 0.22, 0.25	0	0.78
		LDL-C	− 0.06	0.59	− 0.10	− 0.38, 0.09	0	0.33
Toppings	Energy (kcal)	SBP	− 0.13	0.27	− 0.13	− 0.34, 0.10	0	0.27
		DBP	− 0.11	0.36	− 0.11	− 0.32, 0.11	0	0.36
		Urine sodium	− 0.09	0.45	− 0.09	−0.33, 0.16	0	0.43

*Correlation is significant at the 0.05 level (2-tailed)

**Correlation is significant at the 0.01 level (2-tailed)

^║^Quantities of blood test results and nutrients were log-transformed where the test of normality was significant

Risk factors for the development of metabolic syndrome were analyzed using a multivariable logistic regression (Table 6). Fasting blood glucose was the significant biomarker associated with the development of metabolic syndrome (OR 1.42, 95% CI 1.12–1.81). We also found that fat (OR 1.28, 95% CI 1.09–1.89), sodium (OR 1.98, 95% CI 1.05–1.95) and energy (OR 1.09, 95% CI 1.01–1.17) from a la carte meal were significantly associated with the development of metabolic syndrome.

**Table 6:** Multivariable-adjusted odds ratios (95% CI) for metabolic syndrome across different frequencies of nutrients and food group (*n* = 94)

Semi-FFQ	Metabolic syndrome					
	No (*n* = 51)	Yes (*n* = 43)	Crude OR	Adjusted OR	95% CI	P-value
Age (years); median ± (IQR)	39.7 ± (7.5)	38.9 ± (6.5)	1.41	1.14	(0.91, 1.42)	0.247
Gender (Female); *n* (%)	44 (63.8)	25 (36.2)	1.91	1.13	(1.04, 1.42)	0.024*
Smoking (Yes); *n* (%)	4 (40.0)	6 (60.0)	1.52	1.02	(0.11, 4.49)	0.15
Alcohol consumption (Yes); *n* (%)	25 (51.0)	24 (49.0)	1.26	1.07	(0.09, 5.23)	0.724
BMI (kg/m2)	27.4 ± (3.6)	28.9 ± (4.3)	1.1	1.23	(0.85, 1.78)	0.268
HbA1c (mmol/mol)	38.9 ± (19.3)	42.4 ± (16.9)	3.15	1.65	(0.30, 4.05)	0.787
FBG (mmol/L)	5.3 ± (0.5)	5.5 ± (0.4)	1.08	1.42	(1.12, 1.81)	0.004**
Cholesterol (mmol/L)	4.9 ± (0.6)	5.4 ± (0.8)	1.01	1.01	(0.96, 1.05)	0.817
Triglyceride (mmol/L)	1.5 ± (0.2)	1.9 ± (0.1)	1.01	1.01	(0.99, 1.03)	0.32
HDL-C (mmol/L)	1.4 ± (2.1)	1.2 ± (0.3)	0.94	0.98	(0.87, 1.11)	0.758
LDL-C (mmol/L)	2.9 ± (0.5)	3.3 ± (0.2)	1.01	1.00	(0.96, 1.03)	0.789
Nutrients and food group						
Sugar (g); median ± (IQR)						
Fruit	8.5 ± (2.5)	9.4 ± (1.1)	1.03	1.06	(0.36, 1.10)	0.052
Beverage	27.6 ± (2.1)	29.9 ± (4.2)	1.02	1.09	(0.70, 1.17)	0.189
Snack	0.5 ± (0.1)	0.7 ± (0.4)	1.05	1.08	(0.29, 3.42)	0.985
Dessert	1.8 ± (0.6)	1.6 ± (0.8)	1.09	1.04	(0.05, 2.61)	0.324
Fat (g); median ± (IQR)						
Snack	2.9 ± (0.9)	3.4 ± (0.4)	1.05	2.06	(0.74, 7.40)	0.67
Dessert	0.7 ± (0.5)	0.6 ± (0.3)	1.06	1.08	(0.23, 5.99)	0.255
A la carte	5.9 ± (0.7)	8.9 ± (0.4)	1.63	1.28	(1.09, 1.89)	0.031*
Noodles	8.3 ± (0.3)	11.4 ± (5.4)	1.03	1.09	(0.45, 1.69)	0.675
Toppings	8.7 ± (0.9)	11.8 ± (0.9)	1.04	1.07	(0.43, 2.01)	0.845
Sodium (mg); median ± (IQR)						
Dessert	10.2 ± (3.8)	9.9 ± (8.3)	1.00	0.99	(0.98, 1.01)	0.332
Snack	137.1 ± (16.8)	184.2 ± (17.5)	0.99	0.97	(0.86, 1.10)	0.683
A la carte	351.5 ± (12.1)	495.9 ± (19.6)	1.01	1.98	(1.05, 1.95)	0.046*
Noodles	598.6 ± (15.3)	664.8 ± (15.2)	1.05	1.00	(0.99, 1.02)	0.43
Toppings	274.7 ± (21.3)	260.9 ± (35.4)	1.06	0.97	(0.98, 0.99)	0.021*
Energy (kcal); median ± (IQR)						
Fruit	44.8 ± (6.4)	48.8 ± (7.1)	1.05	1.08	(1.01, 1.17)	0.035*
Beverage	190.6 ± (25.8)	214.2 ± (35.8)	1.07	1.02	(0.99, 1.05)	0.17
Snack	56.8 ± (19.4)	74.2 ± (8.6)	1.03	1.01	(0.84, 1.18)	0.988
Dessert	21.3 ± (2.8)	17.3 ± (3.0)	1.10	1.20	(0.90, 1.61)	0.214
A la carte	165.6 ± (17.9)	229.5 ± (23.3)	1.02	1.09	(1.01, 1.17)	0.026*
Noodles	156.3 ± (16.8)	204.6 ± (18.9)	1.01	0.99	(0.94, 1.03)	0.575
Toppings	150.3 ± (12.6)	161.6 ± (15.1)	1.02	1.04	(0.98, 1.11)	0.182

Odd ratios (OR) and their 95% confidence interval for the metabolic syndrome were estimated using multivariate logistic regression models. Mean ± (SD) for all such values, except for variables was determines. Metabolic syndrome defined as the presence of DM-DLP, HT-DLP, HT-DM and HT-DM-DLP

*Odd ratio is significant at the 0.05 level (2-tailed)

**Odd ratio is significant at the 0.01 level (2-tailed)

## Discussion

In this study, the correlations between three critical nutrients for metabolic syndrome, sugar, fat, sodium, and biochemical results were crucial for Thai semi-FFQ validation. We found that some biochemical results increased significantly with an increase in the major nutrients estimated by our semi-FFQ.

Based on the semi-FFQ estimate of sugar intake, most fruits and desserts were significantly correlated with FBG and HbA1c. In 2005, the ATTICA study in Greece set out to find the relationship between diet consumption and blood glucose among people without cardiovascular disease and diabetes. Unfortunately, Panagiotakos et al. did not find a significant association between fruit consumption and FBG, and the correlation coefficient was 0.01 [16]. The correlation coefficient of the ATTICA study was lower than that of our study, which may be due to self-administration. In addition, the nutrient composition of fruit in Greece is different from that of Southeast Asia and Thailand due to climatic and geographical differences.

Another cross-sectional study conducted in East England in 2018 examined the association between dietary sugar from different sources and metabolic markers. The researchers used a semi-FFQ for dietary assessment. They found that sugar from liquids (*r* = 0.16) and free sugar (*r* = 0.07) correlated with plasma glucose [17]. In 2019, the Japan Public Health Center-Based Prospective Study (JPHC) compared two standard methods for validating sugar intake from semi-FFQs: urinary sugar and food records. The study found that sugar intake from a semi-FFQ was positively correlated with dietary records (*r* = 0.34) and urinary sugar concentration (*r* = 0.40). Moreover, the researchers suggested that multiple measurements of urinary sugars lead to a high correlation and are more valuable than single or double measurements [18].

The natural sugars in fruits increase blood glucose nearly as much as sucrose [19]. Tropical fruits, which contain high levels of natural sugar, are widely consumed by Thais because of their digestive assistance and intestinal cleansing properties. It is common practice for Thais to finish their meals with fruits [20]. Moreover, processed foods, snacks, and desserts frequently use high-fructose corn syrup as a sweetener. This processed sugar helps improve the texture of desserts. Some studies did not find a significant difference between the effects of high-fructose corn syrup and sucrose on blood glucose [21, 22]. Other research found that consuming fructose with glucose from starch worsened insulin resistance [23].

As for the relationship between the source of fat intake and biochemical lipid profiles, 8 of the 11 a la carte dishes were correlated with an increase in triglyceride levels and a decrease in HDL-C levels. These a la carte dishes generally contained high amounts of saturated fats, tropical oils and carbohydrates (> 60% of total energy intake). Excessive consumption of both fats and carbohydrates in the Thai a la carte dishes can therefore negative affect triglycerides and HDL cholesterol [24, 25]. According to two previous studies’ findings on the nutritive values and nutrient profiles of mixed dishes [7, 26], the nutrient profiling score for fat in the a la carte group was low. Moreover, almost 50% of the rice-with-topping dishes were related to increased levels of cholesterol and LDL-C.

In 1993, a study in the Netherlands compared three methods: semi-FFQ, diet history, and biomarkers of fat intake. The investigators reported some statistically significant correlation coefficients between fatty acids in erythrocyte membranes and fatty acids from diet. The range of correlation coefficients was 0.15–0.27, which is close to our results. Another study by the ALSPAC (Avon Longitudinal Study of Parents and Children) team in 2001 reported an association between blood lipid levels and dietary intake. Correlation levels ranged between 0.178 and 0.209. The ALSPAC team also reported a correlation between total cholesterol and total fat intake (*r* = 0.209) [27].

Avoiding excess fat became essential for people with MS, particularly, in fat contained in fried dishes in the a la carte and rice-with-topping groups. Typical examples were rice with stir-fried crispy pork and kale, rice with stir-fried pork and hot basil, spicy shrimp paste fried rice, stir-fried crispy catfish with chili paste, stir-fried mixed vegetables with oyster sauce, and stir-fried pumpkin with eggs.

Regarding the correlation between sodium intake and biological results, we found that snacks and dishes from the a la carte group were correlated with blood pressure but not sodium in urine. Some studies found that the correlation between semi-FFQ and 24-h urine sodium was low. Day et al. [28] reported a correlation between semi-FFQ and 24-h urine sodium (*r* = 0.13). This value was similar to those of our study, which found correlations between semi-FFQ and 24-h urine sodium of *r* = 0.12 for snacks, *r* = 0.11 for a la carte dishes, and *r* = 0.08 for noodles. However, research in Brazil reported no correlation between urinary sodium excretion and its semi-FFQ (*r* = 0.18) [29]. Although urinary sodium collection is one of the gold-standard methods to measure the sensitivity of salt intake, a high instability of urinary sodium has been demonstrated [30, 31]. Several factors affect urinary sodium excretion. They include sodium transport mechanisms, sodium absorption, salt sensitivity, micronutrient interactions, and hormones (such as aldosterone and vasopressin) [32]. As for energy intake and biological results, we did not find significant correlations except for desserts with TG (*r* = − 0.262) and cholesterol (*r* = 0.288). The correlation between energy intake and lipid profiles should be positive due to fat consumption. However, supporting data were unavailable.

There were some limitations in our study that affect our results. First, the study did not account for any variations in individual body metabolism and physical activity levels that might have occurred during that period. Second, our study did not find any correlation between the 24-h urine sodium and sodium intake levels, despite this being the gold standard. A review article suggested that at least two to seven 24-h urine collections be performed to increase accuracy [33]. However, because our subjects were medical personnel with typically unpredictable lifestyles, 24-h urine collection tended to be challenging to execute. Third, the investigators realized during their participant interviews that the semi-FFQ was missing some food items that might be important to biochemical results (e.g., alcoholic beverages). Fourth, seasonal fruits and food festivals (such as vegetarian festivals for Thai-Chinese) can affect food availability and hence Thais’ eating behaviors. Therefore, some tropical fruits eliminated from the draft semi-FFQ might have later been available during the investigator interviews with the study participants.

On the other hand, there are some strengths of our study. First, the Thai semi-FFQ used five portion sizes that were based on the standard portion size: − 25%, − 50%, 100%, + 150%, and + 200%. Using several portion sizes helped decrease the effects of variations introduced by individuals’ different portion sizes. Second, food photography clearly illustrated the different serving sizes of mixed dishes, which facilitated the conduct of interviews in the data collection phase in week 4. Third, the inclusion of mixed-dish food items (namely, the rice-with-topping dishes) boosted the effectiveness of our questionnaire and facilitated respondents’ understanding of the food item choices.

## Conclusions

Thai food has a unique characteristic since it often pairs various ingredients and seasoning in one menu. This semi-FFQ is a tool that offers relatively valid ranking for intake of energy, nutrients, single foods, and mixed dishes based on Thai menus associated with a risk for developing metabolic syndrome and NCDs. The validated Thai semi-FFQ could be a reasonable dietary assessment tool for future epidemiological studies in the country.

## Data Availability

Not applicable.

## Abbreviations

DBP: Diastolic blood pressure
DLP: Dyslipidemia
DM: Diabetes mellitus
FBG: Fasting blood glucose
FFQ: Food frequency questionnaires
HbA1c: Glycated hemoglobin
HDL-C: High-density lipoprotein cholesterol
HT: Hypertension
LDL-C: Low-density lipoprotein cholesterol
MS: Metabolic syndrome
NCDs: Non-communicable diseases
SBP: Systolic blood pressure
semi-FFQ: Semiquantitative food frequency questionnaire
TG: Triglyceride
